# Developmental allometry and paediatric malaria

**DOI:** 10.1186/1475-2875-11-64

**Published:** 2012-03-06

**Authors:** Erica MW Billig, Wendy P O'Meara, Eleanor M Riley, F Ellis McKenzie

**Affiliations:** 1 Fogarty International Center, National Institutes of Health, Building 16, Bethesda, MD 20892, USA; 2 Duke University School of Medicine and Duke Global Health Institute, Durham, NC, USA; 3 Moi University, Eldorat, Kenya; 4 Department of Infectious and Tropical Diseases, London School of Hygiene and Tropical Medicine, Keppel Street, London, WC 1E 7HT, UK

**Keywords:** Malaria, Age-dependent, Allometry, Severe malarial anaemia, Cerebral malaria, Paediatric malaria

## Abstract

WHO estimates that 80% of mortality due to malaria occurs among infants and young children. Though it has long been established that malaria disproportionately affects children under age five, our understanding of the underlying biological mechanisms for this distribution remains incomplete. Many studies use age as an indicator of exposure, but age may affect malaria burden independently of previous exposure. Not only does the severity of malaria infection change with age, but the clinical manifestation of disease does as well: younger children are more likely to suffer severe anaemia, while older children are more likely to develop cerebral malaria. Intensity of transmission and acquired immunity are important determinants of this age variation, but age differences remain consistent over varying transmission levels. Thus, age differences in clinical presentation may involve inherent age-related factors as well as still-undiscovered facets of acquired immunity, perhaps including the rates at which relevant aspects of immunity are acquired. The concept of "allometry" - the relative growth of a part in relation to that of an entire organism or to a standard - has not previously been applied in the context of malaria infection. However, because malaria affects a number of organs and cells, including the liver, red blood cells, white blood cells, and spleen, which may intrinsically develop at rates partly independent of each other and of a child's overall size, developmental allometry may influence the course and consequences of malaria infection. Here, scattered items of evidence have been collected from a variety of disciplines, aiming to suggest possible research paths for investigating exposure-independent age differences affecting clinical outcomes of malaria infection.

## Background

In 2009, an estimated 243 million cases of malaria led to approximately 863,000 deaths around the world, 80% of which WHO estimates were in infants and young children [1]. It is widely known and accepted that children are at increased risk for severe disease and death between six months and five years of age. Many studies have attempted to decipher which aspects of the parasite, host, and external environment lead malaria infection to severe disease in some, yet remain asymptomatic in others. Although acquired immunity plays a large role in protection, the host's age, apart from prior exposure, may independently influence the infection's severity. This paper considers the possibility that, for instance, in young children malaria parasites are attacking populations of erythrocytes that are intrinsically smaller, in hosts whose immune responses are intrinsically lower, slower or less durable, and that these features might have clinical correlates.

The *Plasmodium falciparum *parasite life cycle begins when an *Anopheles *mosquito injects sporozoites into the human host. The parasites travel through the bloodstream into the liver, where they invade and replicate, releasing approximately 30,000 merozoites per hepatocyte [2]. The merozoites invade erythrocytes (red blood cells: RBCs). The parasite remains in the erythrocyte for about 48 h, maturing through the ring, trophozoite, and schizont stages, at which point the RBC bursts and releases 8 - 32 new merozoites that invade new RBCs. From the trophozoite stage until it bursts, the infected RBC typically adheres to endothelium and so is sequestered, out of circulation. After a few such cycles, clinical symptoms may begin to appear. A small portion of invading merozoites become gametocytes, the sexual phase of the parasite [3], which can infect a biting mosquito and continue the transmission cycle.

Severe *P. falciparum *infections typically present two distinct clinical manifestations: severe malarial anaemia (SMA) or cerebral malaria (CM). In both, severe disease is generally associated with higher levels of parasitaemia and consequently exaggerated pathogenesis of infection, including rosetting (in which 10 or more uninfected cells clump together around a single infected RBC), cytoadherence, and increased clearance of both infected and uninfected RBCs, discussed in detail below. SMA is associated with high peripheral parasitaemia, low haematocrit, and decreased haematopoiesis [4]. Increasing levels of parasitaemia are associated with decreasing levels of haemoglobin, suggesting a causal relationship between parasitaemia and SMA [5]. SMA in children under five may be more common in boys, although the reason is unknown [6]. Changes in RBCs with host age, such as size, density, overall number, and surface chemical properties may influence pathogenesis. In addition, host factors affecting RBC production and clearance, including spleen structure, may affect anaemia severity.

Despite numerous studies and the identification of several significant contributing factors, the pathogenesis of CM remains somewhat opaque. Although parasitaemia and CM appear correlated, no causal relationship between degree of parasitaemia and CM has been firmly established [7,8]. Many studies point to erythrocyte sequestration in the brain as important, although this has not been observed in all cases. However, the presence of infected RBCs in retinal capillaries is strongly associated with CM [9], and fatalities putatively due to CM, but without erythrocyte sequestration, can be attributed to other infection-related causes [9,10]. Cerebral clinical manifestations may arise from RBC rosettes cytoadhering to endothelium, clogging blood flow to and within the brain [11]. Platelets may have a significant role in the attachment of infected RBCs to the brain endothelium [12]. In addition, it has been noted that CM in children presents differently than in adults. In adults, convulsions are rarely observed, coma arises after a few days of gradual decline, and fatal outcomes are typically due to renal failure, liver failure or pulmonary oedema [13]. In children, coma arises quickly, and there is increased permeability of the blood brain barrier (BBB), raised intracranial pressure, and cerebral oedema [14]. Convulsions are observed in the majority of children with CM, and neurological sequelae are observed more frequently in children than adults [13]. Although the pathogenesis remains unclear, developmental changes may affect the risk for and outcome of CM: propensity for erythrocyte sequestration, cytoadherence, and rosetting may be influenced by RBC size, surface proteins, and deformability. Changes in the size of the brain, cerebral blood flow, and myelination may affect clinical manifestation as well.

Numerous studies have shown that the severity of malaria infection, as well as its clinical manifestations, changes with age. The incidence of severe anaemia and cerebral malaria is higher in children [15-17], and younger children are more likely to suffer SMA, while older children are more likely to develop CM [18-21]. In areas of high endemicity, severe malaria is uncommon after age five, at which point the risk for symptomatic malaria falls significantly as well. Across all levels of transmission intensity, CM appears to be uncommon in children, especially those under four, suggesting the presence of "age-dependent physiological factors that operate independent of acquired immunity" [21]. Thus age differences in clinical presentation may involve inherent age-related factors as well as still-undiscovered facets of acquired immunity, perhaps including rates at which relevant aspects of immunity are acquired [22].

**Figure 1:**
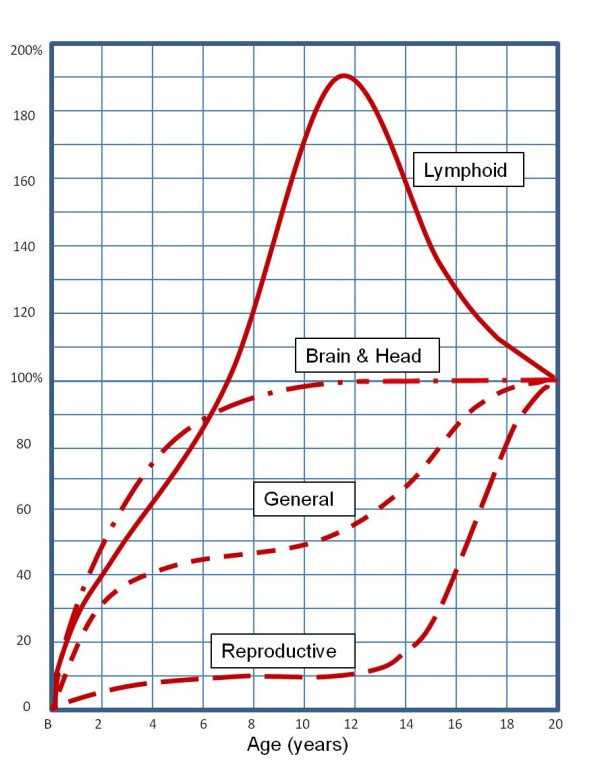
**Developmental growth curves**. Developmental growth curves of different parts and tissues of the human body, each plotted as a percentage of the total gain from birth to 20 years of age (i.e. size at age 20 is 100 on the vertical scale). Height and most body measurements follow the "general" curve. Note that the brain (and the head containing it) develops earlier than any other tissue; at birth it is already 25% of its adult weight, and 90% at age five. Lymphoid tissue reaches its maximum just before adolescence, and then declines to its adult value as the reproductive organs rapidly increase. (Drawn after Scammon, 1930 [25]).

**Figure 2:**
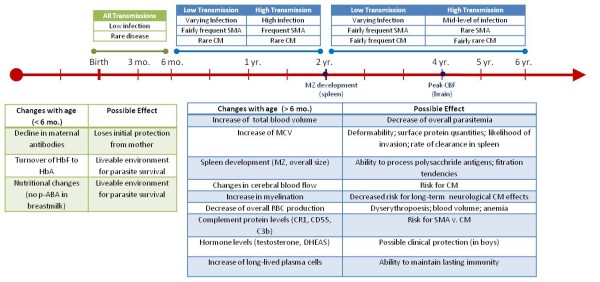
**Summary of the overall changes with age**.

This paper explores the concept of "allometry" - the relative growth of a part in relation to an entire organism or to a standard - as a possible explanation for some age-related differences in malaria infection. Since malaria affects a number of organs, including the liver, red blood cells, white blood cells and spleen, all of which may develop at rates independent of each other and of the child's overall size, each may influence the course and consequences of malaria infection differently at different times. For example, children have a much larger liver mass in relation to body weight than adults, which may influence the rate at which drugs and toxins are metabolized and cleared, and thus the strength of their effects [23]. The non-linear scaling of liver to body mass during development means that paediatric drug dosages calculated as simple fractions of adult dosages, corresponding to relative body mass, could be ineffective or dangerous. Similarly, for drugs cleared through the kidney, it may be important to consider the different rates at which different kidney functions mature: glomerular filtration reaches adult levels at six to 12 months of age, while tubular secretion requires one to five years [24]. Hence allometry is an object of ongoing research and debate in pharmacology and toxicology, as it is in nutrition, evolutionary biology and many other fields (see Additional file 1 and Figure 1). This paper collects scattered items of evidence from a variety of disciplines, aiming to suggest possible research paths for investigating exposure-independent age differences affecting clinical outcomes of malaria infection (as summarized in Figure 2).

### Exposure *vs *age

In areas of high endemicity, children between six months and five years of age develop the most severe clinical response to infection, while in areas of low endemicity, older children and adolescents are most at risk [17]. The first one or two infections are the most severe; particularly in highly endemic areas, age is often used as a surrogate first approximation for exposure, and - in non-fatal cases - clinically protective immunity. Many studies use age as an indicator for both. However, though transmission intensity and acquired immunity must be important factors in age-varying responses [16,17,21,26], some age differences remain consistent over varying transmission levels, apparently independent of previous exposure.

In areas of high transmission compared to low transmission, children develop protection against severe clinical malaria at early ages and less often present with CM, while adults rarely develop symptoms [27]. Separating exposure-dependent from exposure-independent age-related factors is extremely difficult, but a set of epidemiological studies of Indonesian transmigrants attempted to do so [28-30]. Among families relocated from Java, an island with little or no *P. falciparum *transmission, to holo-endemic Irian Jaya, the previously unexposed Javanese children and adults showed unique patterns of malaria symptoms relative to each other and to age-matched locals. In local residents, younger children had more frequent and more severe infections than did adults, as would be expected. The transmigrant children and adults had similar incidence of infection upon initial measurement; however, after three months of residence the prevalence of clinically severe malaria was significantly higher in adults than in children. After one to two years, adult migrants had less frequent and less severe infections than their children who had experienced the same exposure to infection, indicating that they had acquired immunity more rapidly than their children. Thus, apparently, among non-immunes the risk of severe malaria increases with increasing age, but after a period in an endemic area this is offset by rapid acquisition of immunity with increasing age. This result has been supported by other studies suggesting that significant protective factors arise during development, apart from previous exposure [31,32].

### Age-related changes in blood and red blood cells

A number of important and possibly clinically relevant changes occur in blood and red blood cells during infancy and early childhood: a switch from foetal haemoglobin (HbF) to adult haemoglobin (HbA), a shift in the overall age profile of RBCs due to different rates of production and decay, and changes in overall blood volume, cell size and cell surface proteins.

It is difficult to isolate the possible protection of HbF due to potentially confounding variables in infants under six months of age, such as nutritional differences (e.g. nursing) or transferred maternal antibodies, which occur in similar time frames [33]. For instance, there is evidence that breast milk lacks p-aminobenzoic acid, a critical nutrient for parasite survival [33]. Maternal antibodies transferred during gestation have also been shown to contribute to protection of young infants [34].

**Figure 3:**
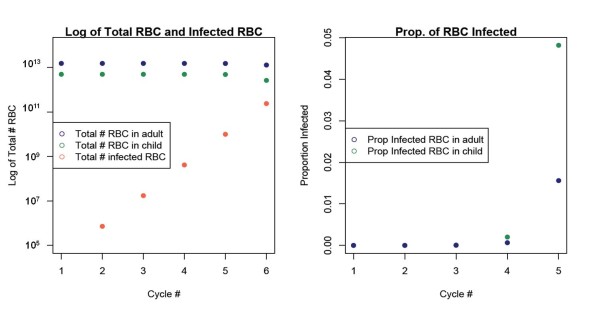
**Percentage of infected RBC**. The percentage of infected RBCs in the total number of RBCs in a child's and adult's body. The total number of RBCs is correlated with average body mass [42,43]. Assuming that the parasite replicates at a constant rate independent of the host, smaller children will have a higher parasitaemia within a given time period. Each cycle is 48 h; therefore cycle 5 occurs 240 h after the parasite is released from the liver.

After this initial period of protection, children in endemic areas become highly susceptible to severe infection. Progression from infection to severe disease and death can be very rapid, particularly in small children. The simple number of available RBCs may be a contributing factor in this progression. Presumably, the biological process of parasite invasion and multiplication in RBCs is independent of the age of the host during malaria infection (i.e. the parasite does not "know" the age of its host). As a child grows, its blood volume grows at a rate correlated with lean body mass [35,36]. Haematocrit and RBC concentration increase slightly with age, suggesting that if the overall blood volume and RBC concentration increases, the absolute number of RBCs must increase as well [37,38]. Thus, as expected, there are fewer RBCs in a small child than in an older child or adult, such that the same number of infected RBCs represents a greater fraction of the total RBC population in a child than in an adult. Although the same number of new host cells are infected during each replication cycle of the parasite, the percentage of host RBCs that are infected must increase more rapidly in a child than in an adult (Figure 3). This idea is supported by observations that younger children develop higher parasitaemia more quickly than older children and adults [39-41]. In a small child the proportion of RBCs infected can increase to more than double that of an adult in just a few days (Figure 3). The smaller pool of available RBCs in a small child and the rapid replication cycles of the parasite shorten the time available to the immune system to respond to the infection, and the time window for treatment, leading to increased severity and fatality of infection.

The rate of RBC clearance increases disproportionately during *P. falciparum *infection: as many as nine to 10 uninfected cells may be cleared for every infected cell, a phenomenon known as the "bystander effect" [44]. It is not known why so many uninfected RBCs are cleared during infection; there are minimal observable surface differences on uninfected cells, but it may be that infection causes antibody sensitization of all RBCs, or that infected RBCs release substances that cause subtle surface changes or reduced deformability of uninfected RBCs [[45,46], and references therein]. Another possibility is that uninfected cells that attach to infected ones are cleared [47]. Since small children have fewer RBCs, and a higher ratio of infected RBCs to uninfected RBCs, this increased rate of clearance removes a larger percentage of the total RBC population, and so may contribute further to the severity of anaemia in younger children [48].

The rate of production of RBCs differs between children and adults. Since young children are growing at a faster rate than adults, and increasing their blood volume in proportion to their lean body mass, they must produce erythrocytes at a faster rate to keep up. At birth, all bone marrow is RBC-producing red marrow, but this proportion continually declines until, in adulthood, only half is red marrow [49]. In infancy and early childhood bone marrow is the main source of RBC production, however after age five production in the long bones steadily declines until puberty [50]. Thus growing children should have a higher proportion of younger RBCs, and *P. falciparum *(as well as *Plasmodium vivax*) preferentially infects young RBCs [51]. Furthermore, malaria has been shown to adversely affect erythropoesis. Though dyserythropoesis has an overall small effect on RBC density [52], its effect on children may be larger due to these developmental factors.

Age-related differences in total cerebral blood flow (tCBF) and regional cerebral blood flow (rCBF) may contribute to observed clinical patterns of CM. tCBF increases drastically within the first few years of life and then gradually declines to adult levels [53]. White matter blood flow peaks between six and 8 months of age, and grey matter blood flow peaks between three and four years of age. The flow to grey matter averages about three times that of white matter, and as a result the overall CBF follows the pattern of grey matter blood flow. At birth, tCBF accounts for about 15% of cardiac output, and peaks around age four, when it accounts for 55% of cardiac output. It then gradually declines to adult levels of 15% [54]. In addition, significant changes in rCBF have been observed with age as different areas of the brain grow [55].

A recent study of fatal paediatric CM cases showed that CM affected both the white and grey matter, although most haemorrhages occurred in the white matter. The increase in CBF with age may predispose older children to clogged blood flow and haemorrhaging. In addition, the majority of cases had a significant increase in brain weight due to oedema compared to that of an uninfected child of the same age, a trend which was not observed in fatal adult cases [56].

Myelination, the process of adding myelin around neural axons, begins before birth and continues through adolescence [57]. Given that paediatric CM is associated with significant damage to myelin [53], it may be that the consequences of this destruction vary with the increasing levels of myelination with age. Although most patients fully recover from CM, long-term effects occur more frequently in children than adults [58]. Children develop symptoms of the central nervous system and most present with convulsions, while adults develop multi-organ failure, and very few have convulsions [58,59]. Although the detailed pathogenesis of CM remains unknown, these age-related changes in CBF, combined with rosetting and brain development, may affect a child's disposition to CM [11].

In addition to the overall change in blood volume and flow, mean red cell volume (MCV) increases with age [15,60]. How RBC size variation affects the parasite's growth and development or clinical manifestations of malaria is unknown, but the RBC size difference may have several consequences. First, cell size may inherently affect invasion probability. Second, assuming that parasite growth is independent of host cell size, cell size may influence internal pressure or the capability for expansion, which affects deformability. When a host is infected with *P. falciparum*, both infected and uninfected erythrocytes lose deformability, exaggerating these effects [61]. Because viscoelasticity has been shown to be consistent between uninfected children and adults, these differences are assumed to be the result of parasite infection [62].

These reductions in size and deformability of RBCs change blood flow dynamics and decrease the ability of a RBC to fit through the splenic filter (see below) [63]. The smaller MCV of young children may influence the deformability of infected RBCs, which may affect splenic filtration, increasing RBC destruction and aggravating anaemia [32,64,65]. Recent research suggests that the spleen is able to clear more cells in younger children, due to their cells' smaller MCV, which may contribute to the increased incidence of SMA in this group [32]. This could be further accentuated during an infection by decreased deformability of smaller infected cells relative to larger RBCs. The increased rate of clearance may lead to an increased clearance of infected RBCs, but may exacerbate SMA due to an increased clearance of uninfected RBC, further reducing the total number of RBCs.

There is evidence to suggest age differences in surface proteins on RBCs, which impact both binding properties and recognition by the spleen [66]. Age-related variations of erythrocyte surface proteins may lead to differences in the ability and tendency of the cells to rosette and cytoadhere, both possible mechanisms for CM [46,67]. Complement receptor 1 (CR1) is a cell surface protein involved in mediating phagocytosis and immune adherence, and has been associated with rosetting. One recent study, across three distinct regions of varying transmission patterns, reported an increase with age in levels of CR1 (CD35), most significantly in young children. This increase occurred at earlier ages and more drastically in malaria-free compared to endemic areas [68]. The increase in CR1 with age may increase a child's susceptibility to CM during growth.

Two other complement proteins, CD55 (decay-accelerating factor) and C3b, are age-dependent and have a significant role as part of the innate immune system during malaria infection [69]. CD55 blocks the membrane-attacking complex, and thus immune destruction [70,71]. CR1 activates C3b, which has many roles including the removal of immune complexes. CD55 inhibits the functioning of the C3 cascade, preventing C3b deposition [69,72]. CD55 levels are lowest in early childhood, and steadily increase through adulthood [73]. C3b levels are highest in early childhood, and decline through adulthood [69]. C3b deposition is associated with increased RBC removal and destruction. Both the presence and density of parasitaemia significantly increase C3b deposition, although the effect of density is not as significant as that of mere presence. The age-associated imbalances of low levels of CR1 and CD55 and high levels of C3b may increase RBC destruction and aggravate SMA in young children [69]. As CR1 and CD55 levels increase in slightly older children, they may increase susceptibility to CM due to their role in rosetting.

The complement cascade, as well as the surface protein CD36, has also been associated with platelet activation. CD36 is present on platelets, macrophages, dendritic cells (DCs), and some endothelium. PfEMP1, a protein expressed by *P. falciparum*, binds to CD36 and is responsible for sequestration of parasites. However, CD36 is not present on brain endothelium and is not responsible for sequestration of parasites in the brain during CM. Rather, it is thought that PfEMP1 binds to platelets, which then secrete cytokines activating the brain endothelium, or, perhaps, themselves bind to endothelium. Some studies have shown an increase in platelet count in the brain during cerebral infection, compared to those without cerebral malaria, possibly due their role in cytoadherence [74]. Thrombocytopaenia is frequently associated with severe malaria infection and disease [75]. It is not clear whether the decrease in platelet count is due to a decrease in production, increased clearance from circulation, or sequestration in the brain and spleen. The specific role of platelets in malaria remains unclear, but there is some evidence relating the severity of thrombocytopaenia with the outcome of infection [76]. Although the "normal" platelet count has a wide range across ages, the accepted normal range is higher in infants and young children than in older children and adults [37]. If platelets are critical for cytoadherence in the brain and are a rate limiting step in sequestering parasites in the brain capillaries in very young children, increased platelet count with age may increase susceptibility to CM.

In addition to the cells themselves, blood vessels may change in size or chemical properties with host age, influencing blood flow, cytoadherence, and parasite clearance rate. For example, there is evidence that levels of VCAM-1, a cell-adhesion molecule important in malaria, decrease throughout childhood, but research on such changes across ages is still sparse [77].

In summary, HbF, small MCV, inherent size differences (total blood volume and blood vessels) and surface concentrations of CR1, CD55, and C3b that change during infancy and early childhood may increase susceptibility to SMA. These same size differences and changes in CBF and platelets may increase susceptibility to CM with age.

Finally, there may be interaction between clinical features of SMA and CM. SMA might provide some protection against CM by reducing the number of RBCs in circulation. If CM is significantly related to rosetting, a reduction in the number of total RBCs, and thus their concentration, would reduce the probability of the cells contacting each other and forming a cluster [46,78]. Although many studies show co-occurrence of CM and SMA at expected levels [40], significant negative association has also been observed [79]. Patients with low packed cell volumes have a significantly lower frequency of presenting with CM than those with higher packed cell volumes [80].

### The spleen

The spleen filters RBCs, and destroys them if infected or senescent. The spleen clears RBCs through two distinct structural mechanisms: physical selection and cell-cell interaction. The spleen is composed of the red pulp, where mechanical filtration occurs, and the white pulp, where most contact with immune-system cells occurs [81]. The spleen also contains the marginal zone, the region bordering the red pulp and white pulp, which contains macrophages especially important for targeting polysaccharide-encapsulated bacteria [82].

To filter RBCs, the spleen uses both fast microcirculation, a closed vascular system, and slow microcirculation, an open system in which RBCs enter a large pool and must filter through the cords in the red pulp and the endothelial cells. Ten to 20% of RBCs are continuously filtered through slow microcirculation; their successful passage depends on the deformability of the cell [32]. RBCs containing HbA typically circulate for about 120 days, changing physically and chemically with age in ways that affect predisposition for splenic clearance. In older RBCs, the volume of water decreases, but the haemoglobin level remains stable, increasing the density and decreasing deformability [83]. During malaria infection, RBCs exhibit these same changes [63]. As parasites grow, the deformability of the cell decreases. So, in the slow, open microcirculation it may be that the spleen retains and destroys infected RBCs due to this change in density [84]. Malaria infected erythrocytes may also be "pitted" in the slow microcirculation of the spleen [85]. Pitting removes the parasite from the cell, and then returns the cell into circulation.

Foetal RBCs have a shorter lifespan due to differences in haemoglobin chemistry, enzyme concentration, and surface proteins [86] that may make them more susceptible to splenic clearance. It is possible that the initial size and haemoglobin composition of the cell affects changes in deformability. The smaller MCV and higher content of HbF of young children may be a factor in the increased frequency of SMA. The spleen may clear more infected and uninfected cells as a result of this initial size difference, and the consequently exaggerated reduction of deformability during infection.

Splenomegaly is common in malaria infection, sometimes extreme to the point of rupture, and has been associated with increased rates of clearance of infected erythrocytes in mice [87,88]. The physical limitations of swelling are unknown, but it is possible that initial spleen size, correlated with age, affects the maximum rate of infected RBC filtration.

Structural differences between infant and adult spleens may contribute to differences in clinical response to infection. Both the marginal zone and red pulp seem to take part in the clearance of infected RBCs, but the marginal zone only begins to appear about 8.5 months after birth, and is not fully developed in human infants until age two [89,90]. The marginal zone B cells are known for their role in T-independent responses, and young infants cannot produce T-independent (TI) antibody responses, possibly due to this immature architecture and an absence of natural memory B cells (discussed below) [90]. However, at least in mice, marginal zone B cells also produce IgM within a few days of antigen presentation [91]. In spleens from splenectomized humans, B cells in the marginal zone also produced IgM. However, there were few IgM-only producing B cells, although more in children under five years of age [92]. Immaturity of the spleen has been noted to cause observable differences in the immune responses of young children, especially in response to polysaccharide antigens, and is likely to influence responses to malaria infection.

### The immune system

A host of any age responds to infection by attempting to destroy the multiplying parasite, but the capacities of immune and other response systems may be more limited in infants. The immaturity of the immune system in neonates is widely recognized, in general terms [93-96]. Young infants do not respond well to T-independent polysaccharide antigens, and create a weaker response than older children and adults to T-dependent protein antigens [93]. This observation may be due to the lack of a marginal zone in the spleen in young children. Age-related differences in immune responses can be observed on a clinical level, for instance in the greater susceptibility of younger people to environmental toxins [97]. Specific causes for these observations remain unknown, but some developmental differences have begun to be recognized.

Though some vaccines given to newborns and young infants work well, others do not elicit effective, durable protection, presumably due to the presence of passively transferred maternal antibodies, weak and abbreviated antibody responses and developmental differences in T-cells and antigen-presenting cells. There seems to be some contribution of both genetics and environment in the patterns of early cytokine response [98]. When Meningococcus C and diphtheria vaccinations are given, even multiple times, the resulting antibody titer declines much more rapidly in infants than adults [99]. As another example, a study in lab mice that compared six vaccines containing the same antigen but different adjuvants found that the most effective in infant mice was least effective in adults [93]. These specific examples indicate that there is some overall immune immaturity, the specifics of which remain unknown, that may also affect infant responses to malaria infection.

*Plasmodium *infection triggers both innate and acquired immune responses. Because the ratio of naïve cells to memory cells declines as the human matures, due to cumulative foreign antigen exposure, it is difficult to differentiate the effects of age per se and previous exposure on these responses [100]. Exposure to malaria also stimulates memory cells that protect the body from future encounters with the same antigen. However, because not all malaria infections become symptomatic, whatever the age of the host, scientists cannot rely on clinical signs as an indication of exposure, and must instead undertake proactive studies of serology.

### Cell-mediated response

The thymus loses its functionality with age, slowing T cell production and differentiation. In fact, the rate of T cell production peaks in early childhood. Maximum thymic cellularity occurs between six months and one year of age, at which point it begins a slow, steady, decline [101,102]; thymic functionality declines at a much faster rate around puberty [101,102].

The immune system includes Th1 CD4+ T cells and Th2 CD4+ T cells among several other T cell subsets. T regulatory (Treg) cells control the differentiation and function of T cells, regulated by the transcription factor Foxp3, as well as the cytokine environment [103]. A balanced capability to produce both Th1 and Treg responses is essential to control malaria infection. Infants do not mount sufficient Th1 responses to infections in general, however, which creates a skewed response. It is thought that the Th1 response is restrained until the capacity to make T regulatory responses has developed, protecting the infant from potentially dangerous runaway inflammatory immune reactions [103]. The Th2 bias is due to some aspect of the regulatory network that is immature, perhaps influenced by passively transferred maternal antibodies [98]. Infants have a higher concentration than adults of recent thymic emigrants (RTEs) circulating in the periphery [104]. As a growing host is exposed to common pathogens over time, the repertoire of circulating CD4+ T cells expands. Increasing numbers of memory T cells with increasing age may increase the potential for "cross-reactive" bystander activation of Th1 cells, leading to more inflammation and thus more CM. As the regulatory responses develop into later life, this balance may be redressed, reducing the risk again [105].

Neonates and young infants produce a Th2 response to antigens but gradually shift to a dominant Th1 response with age [106]. Both too much and not enough Th1 response are associated with severe disease [106,107]. Though the reasons for the Th2 bias in infants and young children remain unknown, the immaturity of their DCs may be a key factor [108,109]. Recent evidence indicates that DCs release cytokines recruiting Th2 memory cells upon activation [110]. DC populations are smaller in infants than adults both in absolute terms and relative to their smaller size [111]. The number of circulating myeloid DCs and plasmacytoid DCs was significantly lower in 12-month olds compared to adults [111]. Infants have a much higher proportion of immature DCs than adults, as indicated by studies of T-cell activity [111].

The inflammatory cytokine TNF-α is associated with cerebral malaria, and it is possible that the bias against producing a Th1 response in young children - whether it is due to inherent age-related properties or exposure and environment - helps to protect them from developing cerebral malaria [93,109]. Exposure to malaria causes infants and young children to produce relatively heightened Th1 responses for their ages, which may help to explain the slightly earlier age of observed cerebral malaria cases in holo-endemic regions [22].

### Humoral response

The Th2 CD4+ T cell bias in infants influences the proportions and levels of cytokines produced, and may affect which antibodies are produced in response to an infection by influencing different B cells [28]. Typically, immunoglobulin M (IgM) is the primary antibody, produced during first exposure to an antigen, and IgG is produced upon subsequent exposure. The baseline concentration of IgG in the blood, nominally in the absence of infection, increases throughout childhood and adolescence [112].

The subclass of IgG response - IgG1 or IgG3 - varies, but the cause of this variation remains unclear. Some research indicates that in malaria the subclass varies by *P. falciparum *antigen. But there may be more involved than the antigen presented. One study showed that children trigger an IgG1 response to the MSP-2 (merozoite surface protein-2) antigen, while their maternally-derived antibodies to MSP-2 were IgG3 [113]. Infant B cells have been shown to produce quantitatively lower IgG and IgM responses than adults to "protein or conjugate vaccine antigens" [93]. Infants have also been shown to produce a lower response to most T-dependent antigens; although the diminished response is most evident in TI antibodies and IgG subclass IgG2, it has also been observed in IgG3 [114].

The IgG3 response to MSP-2 serogroup A is highly correlated with clinical immunity to *P. falciparum*, so the possibly diminished antibody response and IgG1 bias in children might be involved with increased severity of disease in infants and young children [115]. However, these studies do not distinguish whether previous exposure or developmental immaturity cause this shift in antibody quantity and subclass.

Another recent study found that adult non-immune travelers had a strong IgG1 (rather than IgG3) response compared to immune adults during infection, suggesting that previous exposure is a significant factor in determining the IgG1 vs. IgG3 response [116]. However, there are still differences by age in the *rate *of acquisition of the different antibody subclasses [116]. Recent research has begun to try to differentiate the acquired responses to *P. falciparum *based on age and the intensity of transmission. One study looked at the ratio of IgG1/IgG3 response across four different levels of transmission, and showed that age and endemicity both independently increase IgG1 subclass at younger ages and increase IgG3 with age for anti-MSP-2 antibodies [117].

**Figure 4:**
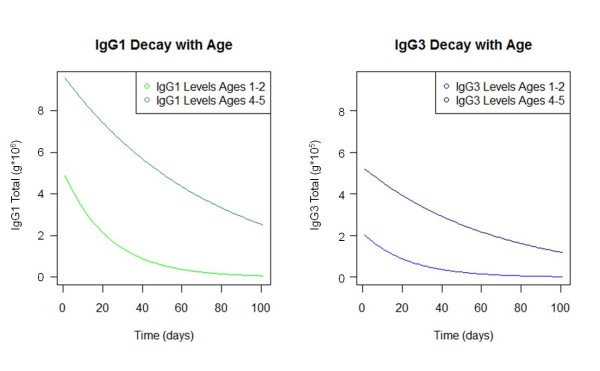
**Rate of antibody decay**. Children of different ages have different initial levels of antibodies, and the antibodies decay at different rates [43,118,121].

There is evidence that the *persistence *of antibodies, as well as the rate of production, increases with age. A recent study in the Gambia found that the half-life of antibodies to an array of *P. falciparum *antigens in older children (ages four to six) was three times that of younger children (ages one to three) during and after malaria infection [118]. Similarly, a study in Kenyan children found that the half-life of their antibodies was much shorter than in healthy adults, and yet another found that the quantity of these same antibodies declined in all age groups, but to about half the level in the younger age groups within the same time interval [119,120]. The differing initial levels of antibodies and differing rates of decay with age lead to significant differences in antibody level over time, following initial antigen exposure (Figure 4).

This concept of varying half-lives is further supported by evidence that IgG levels in children have been shown to fluctuate seasonally, while the levels of antibodies in adults remained much more stable [119,122]. This difference may be caused by shorter-lived plasma cells, so that the child's recognition of an antigen does not persist as long as an adult's, and consequently the plasma cells fail to continue secreting IgG.

Long-lived plasma cells are necessary to provide an extended effective response to (non-chronic) infection, but are apparently lacking in young children [93,123], as they are in infant mice [124]. Both long-lived and short-lived plasma cells produce antibodies, but it is the long-lived plasma cells that sustain levels of antibodies during periods of little or no exposure to infection. Memory B cells are potential plasma cells, pending repeat exposure to antigen; long-lived memory B cells function for long periods of time independently, and are needed to renew the pool of short-lived and long-lived plasma cells. The duration of antibody response after a single exposure may provide insight into which plasma cells are secreting the antibodies. Recent evidence suggests that malaria disease may induce changes in the levels of B-cell activating factor (BAFF), which has an important role in B-cell differentiation and the maturation of long-lived plasma cells, possibly further complicating the development of immune responses in young children [125].

**Figure 5:**
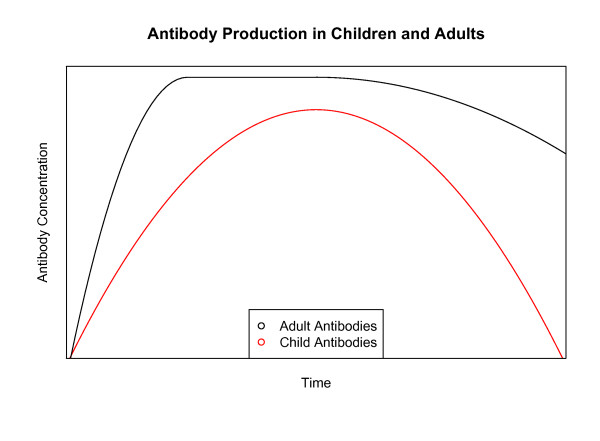
**Antibody production in children and adults**. A conceptual diagram of antibody levels in children and adults. First, the rate of production is slower in children than adults. Then, the total overall amount produced is lower in children than adults. Finally, the antibodies decay at a faster rate in children than adults. Combined, these effects may have a significant impact on the ability of children to target *Plasmodium falciparum *antigens.

Thus, the combination of different rates of antibody production and decay with age, as well as the absolute amount of antibodies produced by plasma cells, may cause significant differences in the quantity of antibody over time, independently of effects from previous exposure (Figure 5).

## Conclusion

This review focuses on the observation that infants, young and older children, adolescents, and adults each show markedly different susceptibility, clinical manifestations, and morbidity and mortality with respect to malaria. While exposure, and thus acquired immunity, may vary with age, studies suggest that a number of independent age-related factors may help to explain these differences.

Malaria affects many organs, each of which play a role in the protection from, development of, and presentation of malaria infection. Well-documented developmental changes in immunity may play a major role in the susceptibility of infants and young children to malaria, but other developmental changes in the liver, spleen, blood cells and blood flow occur at ages that correspond to noted changes in malaria susceptibility and manifestations. The relative impact of these anatomic and physiologic developmental changes, alone or in combination, has not yet been systematically examined. However, if the aim of a malaria vaccine is to shift the response of a young child to that of an older child - i.e. to minimize the frequency and severity of clinical episodes - then expectations must be calibrated to allow for the effects of development that arise with age per se, as well as with exposure.

## Abbreviations

WHO: World Health Organization; RBC: Red blood cell; CM: Cerebral malaria; SMA: Severe malarial anaemia; BBB: Blood-brain barrier; HbF: Foetal haemoglobin; HbA: Adult haemoglobin; tCBF: Total cerebral blood flow; rCBF: Regional cerebral blood flow; MCV: Mean cell volume; CR1: Complement receptor 1; DC: Dendritic cell; TI: T-cell independent; TRE: Recent thymic emigrants; IgG: Immunoglobulin G; IgM: Immunoglobulin M; MSP: Merozoite surface protein.

## Competing interests

The authors declare that they have no competing interests.

## Authors' contributions

EB conducted the literature review. All authors wrote, read and approved the final manuscript.

## Supplementary Material

Additional file 1 **What is allometry? **[126-132]. Click here for file
