# Supplementary material for: Developmental allometry and paediatric malaria
Source: Malar J. 2012 Mar 6;11:64. doi: 10.1186/1475-2875-11-64 (PMC3331816; doi:10.1186/1475-2875-11-64)
Supplement: Additional file 1 — What is allometry? [126-132]. [file 1475-2875-11-64-S1.PDF]

Allometry denotes "growth of a part at a different rate from that of [the] body as a whole or of a standard" [126]. The special, linear case in which the rates are identical is isometry. The usual, non-linear relationship is conventionally described by an equation of the form $y = bx^a$, where y is the part, x the whole or standard; the values of a (the "allometric exponent") and b (the "allometric coefficient") are empirically derived constants. For instance, a recent analysis of 51 species of primate (excluding humans) relates brain and body weight as $(\text{brain}) = (\text{body})^{0.761}$. Plotted on a log-log chart, this equation is the straight line [log brain (g)] = 1.24 + 0.761 [log body (kg)], for which $r^2 = 0.92$ [127]. In human post-natal development, growth of the heart scales isometrically with that of the body as a whole, while the head starts much larger and becomes proportionally smaller as individuals grow to their final body sizes [128].

Allometry applies to physiology as well as to anatomy, and to their intersection, most famously to the relationship between basal metabolic rate (BMR) and body mass. "Kleiber's law" [129] holds that BMR = b*(body mass)^0.75, for the "mouse-to-elephant" curve across mammals, and for many other taxa. This sort of allometry can be important in evaluating effects of nutrition and exercise, and of pharmaceuticals, in scaling from animal models to humans and in scaling paediatric dosages. There is continuing debate about exact values of coefficients and exponents, appropriate methodologies and taxonomic levels, and underlying structural and functional factors that might give rise to such widespread scaling at the (roughly) three-quarters power [130-131].

Here, the concept of allometry is applied broadly, to parts or processes that mature in children and reach adult status at different rates (Figure 1). To the best of our knowledge, this is only the second paper to apply allometry to infectious disease. The first [132] suggested that disease progression in infections of birds and mammals in general scales with the body size and BMR of the host species, at least partly in line with several of our suggestions about how, on an individual-developmental scale, constraints of architecture and/or process might help to explain some age-related differences in human malaria severity.
